# How do essential oil composition and phenolic acid profile of *Heracleum persicum* fluctuate at different phenological stages?

**DOI:** 10.1002/fsn3.1916

**Published:** 2020-09-28

**Authors:** Saeid Hazrati, Saeed Mollaei, Hossein Rabbi Angourani, Seyyed Jaber Hosseini, Mojde Sedaghat, Silvana Nicola

**Affiliations:** ^1^ Department of Agronomy Faculty of Agriculture Azarbaijan Shahid Madani University Tabriz Iran; ^2^ Phytochemical Laboratory Department of Chemistry Faculty of Sciences Azarbaijan Shahid Madani University Tabriz Iran; ^3^ Research Institute of Modern Biological Techniques University of Zanjan Zanjan Iran; ^4^ Department of Agronomy Faculty of Agriculture Tarbiat Modares University Tehran Iran; ^5^ Department of Environmental & Plant Biology Ohio University Athens OH USA; ^6^ Department of Agricultural, Forest and Food Sciences, DISAFA, Vegetable Crops & Medicinal and Aromatic Plants, VEGMAP University of Turin Turin Italy

**Keywords:** cinnamic acid, flowering stage, octyl acetate, Persian hogweed, phytochemical composition

## Abstract

*Heracleum persicum*, commonly named Persian hogweed, is a principal native medicinal plant in Iran. Collecting *H. persicum* at the most appropriate growing stage is the key factor to achieve the high phytochemical quality to meet consumer's needs. In the present experiment, the aerial parts of this plant were harvested at up to six different developmental stages during the growing season to determine the phytochemical profiles. Our results indicated that the highest essential oil content was obtained in the mid‐mature seed stage (3.5%). The most elevated extract content was recorded in the floral budding stage (10.4%). In the vegetative stage, limonene (18.1%), in floral budding stage, caryophyllene (14.1%), anethole (14.6%), and β‐bisabolene (12.7%), in the full flowering stage, myristicin (15.0%), and hexyl butyrate (9.1%), in the early development of seeds stage, hexyl butyrate (32.1%), and octyl acetate (11.7%), in the mid‐mature seeds stage hexyl butyrate (38.8%), octyl acetate (14.5%), in the late‐mature/ripe seeds stage, hexyl butyrate (23.6%), and octyl acetate (10.5%) are recorded as the main components. The highest phenolic acids content was obtained in the floral budding stage (287.40 mg/g dried extract). The analysis of phenolic acids demonstrated cinnamic acid (8.0–225.3 mg/g extract), p‐coumaric acid (1.7–39.2 mg/g extract), p‐hydroxybenzoic acid (0.8–16.8 mg/g extract), and ferulic acid (2.4–15.8 mg/g extract) as the main phenolic acids. Cinnamic acid was found as the major phenolic compound in the vegetative stage following by floral budding, the full flowering stage, the early development of seeds, and late‐mature/ripe seeds stages. P‐coumaric acid was the most abundant phenolic compounds in the mid‐mature seeds stage. In this regard, the harvest time of *H. persicum* aerial parts can be selected to achieve the highest secondary metabolites of interest. The results of this study can be used as a guideline for grower to obtain the highest possible amount of desirable metabolites, beneficial in both food and pharmaceutical industries as well as their undeniable economical benefits.

## Graphical abstract

The development stage has a significant impact on the content and composition of both essential oil and phenolic acid composition. The harvest time of H. persicum aerial parts can be selected to achieve the highest secondary metabolites of interest.

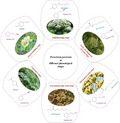

## 1 INTRODUCTION

Until now, more than 125 species of the genus *Heracleum* have been discovered all around the world. Most species of this genus are distributed in Asia which among them ten perennial aromatic species grow in the flora of Iran. *Heracleum persicum* L., commonly known as Persian hogweed simply hogweed, or Golpar, is a polycarpic perennial herbaceous and a flowering shrub belongs to Apiaceae family. It is originally native to humid mountainous areas of Iran, Iraq, and Turkey (Radjabian et al., 2013).

This plant is widely distributed and grown in Iran in regions with different ecological conditions. The best‐growing condition for this plant is moist and fertile areas, especially in the mountains in the northern part of the country, with altitudes from 1,500 to more than 3,000 m above the sea level. (Hoseinifar et al., 2016; Radjabian et al., 2013).

*H. persicum* L. is a perennial herb that usually grows up to 1.5–2 m. This alternate‐leaved plant has an anise‐like smell with thick and hollow stem. The leaf blades are elongated, densely haired on the lower side, glabrous on top, and pinnate with blunt‐toothed margins. The flowers with five petals and five stamens are small with pale white and lime‐green color. The fruits are broadly obovate, with slightly ridged schizocarp (Majidi & Sadati Lamardi, 2018).

Different parts of this plant have a long reputation as a natural remedy in the Iranian folk medicine. Aromatic fruits of *H. persicum* are extensively used in the daily diet of the general Iranian population as a flavoring agent and spice and the stems are used in making pickles. In addition, in Iranian traditional medicine, its fruits are used as a carminative, anti‐inflammatory, digestive aid, antimicrobial tonic, and antiepileptic. It is also believed that this plant can work against stomach ailments, flatulence, infections, memory impairment, forgetfulness, vertigo, and stupidity aphrodisiac (Davari & Ezazi, 2017; Majidi & Sadati Lamardi, 2018; Panahi et al., 2011; Sefidkon et al., 2004). According to previous studies, *H. persicum* have been shown several biological activities, such as antioxidant (Çoruh et al., 2007; Firuzi et al., 2010), anti‐inflammatory, analgesic (Hajhashemi et al., 2009; Majidi & Sadati Lamardi, 2018), antidiabetic (Dehghan et al., 2016), antihyperlipidemic (Dadjo et al., 2015; Hajhashemi et al., 2009), cardioprotective (Panahi et al., 2013), gastroprotective (Majidi & Sadati Lamardi, 2018), neurological (Sayyah et al., 2005), immunomodulatory (Sharififar et al., 2009), hepatoprotective (Majidi & Sadati Lamardi, 2018), antibacterial (Shariatifar et al., 2017), antifungal (Khosravi et al., 2016; Sadeghi Nejad et al., 2014), anticonvulsant (Sayyah et al., 2005), and insecticidal properties (Izakmehri et al., 2013). Recently, different phytochemical compounds such as tannins, saponins, alkaloids, flavonoids, and furanocoumarins were extracted from different parts of *H. persicum* plants (Razzaghi‐Abyaneh et al., 2013). Among them, the essential oil composition of *H. persicum* fruits has been widely studied, and the results suggests that the fruit can be considered as a suitable source of essential oils and aliphatic ester compounds (Amanpour et al., 2016; Firuzi et al., 2010; Gharachorloo et al., 2018; Radjabian et al., 2013, 2014; Scheffer et al., 1984). Until now, the majority of studies on *H. persicum* have focused on its essential oil fraction. Limonene, γ‐terpinene, anethole, hexyl butyrate, octyl acetate, hexyl‐2‐methylbutanoate, hexyl isobutyrate were identified as the major constituents of the *H. persicum* essential oil. The amount of essential oils constituents depends on the plant part where essential oil is extracted (Davari & Ezazi, 2017; Gharachorloo et al., 2018; Radjabian et al., 2013; Sefidkon et al., 2004).

The major source of secondary metabolites in plants of the Apiaceae family is the leaves, stems, flowers, and especially fruits. Secondary metabolite contents are mostly allocated to the essential oils and phenolic compounds, which are more important for industrial use, than other phytochemical compounds. Other favorable compounds with antioxidant properties are mainly used in the food industry, while essential oil is edible and has pharmaceutical properties (Tajkarimi et al., 2010). Medicinal plants with antioxidant properties can replace synthetic antioxidants, because they are less costly and more environmentally friendly. In addition to the above‐mentioned advantages, dietary phenolic antioxidants of *H. persicum* play important roles in delaying the development of chronic diseases such as cardiovascular diseases, inflammatory bowel syndrome, and Alzheimer's diseases (Goleniowski et al., 2013; Kaurinovic & Vastag, 2019). Studies have shown that *Heracleum* species are rich in phenolic compounds and exhibit high biological activities (Firuzi et al., 2010) making them worthy medicinal plants to study.

According to previous researches, phenological and harvest stages are bare bone essential factors that influence phenolic compounds contents as well as their biological activities in different organs of medicinal plants(Esmaeili et al., 2018; Grevsen et al., 2009; Hazrati et al., 2019). Choosing the best‐growing stage will help us to harvest the higher amount of essential oil yield as well as other beneficial compounds. Moreover, the amount of phenolic compounds in medicinal plants is affected by genetic variation among different species, even within the same species and also by the maturity of plant organs at harvest time (Alirezalu et al., 2018; Deveci et al., 2018; Hazrati et al., 2019; Li et al., 2020; Wang et al., 2009). However, the proper time of harvest to achieve the maximum beneficial compounds of *H. persicum* remains unknown and needs more research work. Until now, there is limited information on phenolic compounds of *H. persicum* in different organs and different growth stages. Thus, it is essential to specify more precisely the time when *H. persicum* plant should be harvested to attain the highest possible quality and quantity of beneficial contents. In this study, we aimed to investigate the distribution pattern of phenolic compounds and essential oil in the *H. persicum*. We also tried to find the best phenological stage that has the high concentration of these components in the aerial parts of *H. persicum* that were collected from the Iranian population at different developmental stages. To the extent of our knowledge, this is the first report aimed to evaluate the phytochemical composition of different growing stages of *H. persicum*, during its biological cycle, which results will be useful to find the best harvest time to reach the high beneficial yield with less harm to the environment.

## 2 EXPERIMENTAL PROCEDURE

### 2.1 Plant material

**Figure 1:**
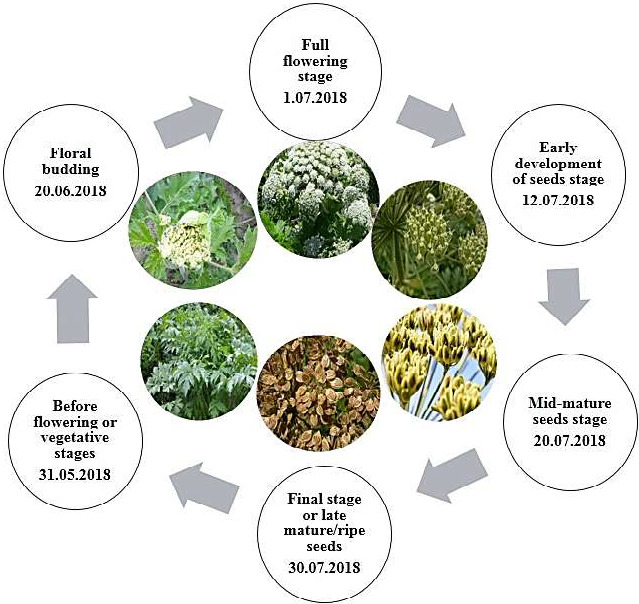
*H. persicum* at different phenological stages

The current study took place in the spring and summer of 2018 in the city of Sarab, East Azerbaijan Province, Iran (Location: 37°56′27″N and 47°32′12″E; altitude: 1,750 m). The first sampling started 30 days after the onset of the vegetative growth, in spring. All samplings were randomly selected from ten *H. persicum* plants. The harvest was done at different developmental stages (Figure 1). Each sampling was repeated three times, and sample's weight was 1 kg per replication. The samples were dried in a shade at room temperature (25°C).

### 2.2 Extraction of essential oils

Applying hydro‐distillation for three hours, the essential oil was extracted from 100 g dried samples in 600 ml of distilled water in a 2‐L flask using Clevenger apparatus extraction technique in three replications (British Pharmacopoeia, 1988). The obtained essential oil was dried over anhydrous Na_2_SO_4_ and kept at −20°C until further analysis.

### 2.3 Preparation of the extracts

In order to prepare the extracts, dried powdered of different samples of *H. persicum* were used. About 30 ml of the methanol was added to 2 g of samples and put in an ultrasonic bath (Frequency, 100 kHz; power intensity, 160 W; temperature, 35°C) for 30 min. Then, the extract was filtered, evaporated, and stored at –20°C until further analysis.

### 2.4 Extraction of phenolic acids

The phenolic acid extraction was performed using our previous work method with some modification (Hazrati et al., 2019). Briefly, 10 ml of 80% ethanol was added to 1.0 g of the dried powdered plant, vigorously shaked, and centrifuged at a speed of 12,000 rpm for 10 min. The collected supernatant was evaporated and stored at –20°C for further analysis.

### 2.5 Analysis of phenolic acids

In order to analyze the phenolic acid compounds, HPLC (Waters 2695, USA) system equipped with a diode‐array detector, a 20 µl loop, and an ODS column (250 mm × 0.46 mm, 5 µm) was operated. The reverse‐phase separation was done with gradient elution solvent A [methanol TFA (99.9:0.1, v/v)] and B [water TFA (99.9:0.1, v/v)] at a flow rate of 0.5 ml/min with the following elution gradient: 20% A, at 0 min; 30% A, (from 0 to10 min); 60% A, (from 10 to 30 min); 80% A, (30 to40 min); 100% A, (40 to 45 min); 20% A, (from 45 to 52 min); isocratic, 6 min. The phenolic compounds were detected at 254 nm, 275 nm, and 320 nm, and identified based on the retention time and spike method. Finally, the standard external method was applied to quantify the studied phenolic acids. The results were expressed as mg per g of the extract weight.

### 2.6 Gas Chromatography (GC)

Essential oils analysis was done using a gas chromatography device (model: Agilent 7890 A G). The separation was carried out using the Column MS HP‐5 (30 m × 0.25 mm, 0.25 µm). The oven temperature program was set at 50°C (3 min) to 260°C with a ramp‐up of 3°C/min and then held for 5 min. The detector and the injector temperature were adjusted at 270 and 240°C, respectively. Nitrogen was used as carrier gas at a rate of 1 ml/min.

### 2.7 GC‐MS chromatography

In addition to GC, a gas chromatography device connected to a mass spectrometer (Model: Agilent 7890 A G Chromatograph and Agilent 5975 c Mass), called GC‐MS chromatography, was used to analyze the essential oil. In this experiment, GC‐MS chromatography was equipped with HP‐5 column (30 m × 0.25 mm, 0.25 µm). According to the planned program, the oven temperature remained constant at 50°C for 3 min and remained constant for 5 min after increasing the temperature to 260°C with a ramp‐up of 3°C/min. Here again, nitrogen was utilized as carrier gas at the rate of 1 ml/min. The analysis was performed with scan time (30 m/z), range of analysis (600 m/z), ionization 0/6 s, 70 Electron volt and solvent evaporation rate of 2 min. The compounds were then identified by index (ki) and Wiley Library and Nist11.

### 2.8 Statistical analysis

In order to determine the relationship between different phytochemical compounds, variance analysis, comparison of means, principal component analysis (PCA), and cluster analysis were done using the statistical package SAS 9.4. All determinations were conducted in triplicate, and the results were calculated as mean value ± standard error (SE). The variations (standard error of means, SE) and the significances of treatment effects (*F*‐test) were calculated and tested using the General Linear Models procedure of SAS. The mean values of treatments were separated by *Tukey's* test (*p* < .05).

## 3 RESULTS AND DISCUSSION

### 3.1 Content of essential oil and extracts

**Figure 2:**
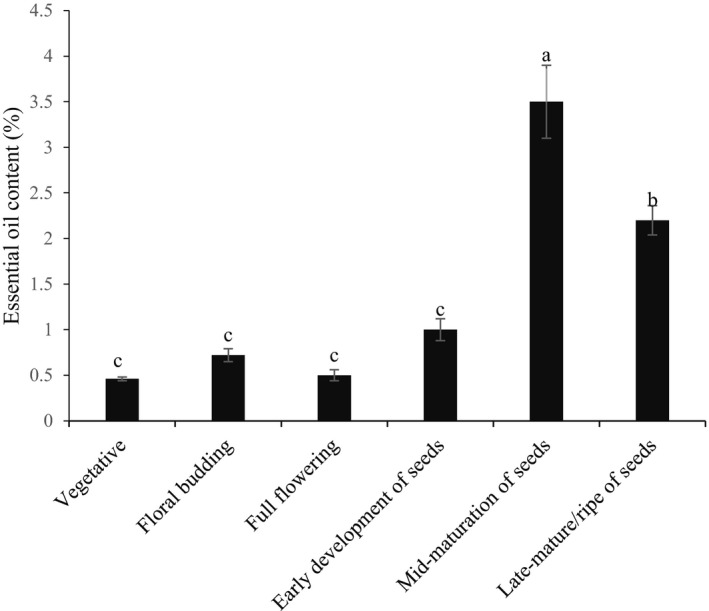
Changes in essential oil content (% w/w) of *H. persicum* at different phenological stages. Essential oil content with different subscripts was significantly different at *p* < .05 (*Tukey's* test)

Medicinal plants have different capacities to produce essential oil at different phenological stages (Li et al., 2020). To achieve their goal, breeders must consider the proper harvest time to reach the best yield for their target purposes, such as pharmaceutical, food, and cosmetic industries applications. For this reason, the essential oil content was studied in different phenological growth stages of *H. persicum*. Statistical analysis of this experiment showed that the essential oil content was significantly different in various phenological stages of this plant (Figure 2). The results indicated that the obtained essential oil yield in the vegetative stages, floral budding stage, full flowering stage, early development of seeds stage, mid‐mature seeds stage and, final stage, or late‐mature/ripe seeds stages were 0.45, 0.72, 0.50, 1.00, 3.50, and 2.20%, respectively. According to these data, the greatest yield was observed at the mid‐development stage of the seeds, while the minimum yield was associated with the vegetative stages (Figure 2). Other medicinal plants such as the *Apiaceae* family, including *Oliveria decumbens* (Esmaeili et al., 2018),*Trachyspermum Ammi* (Soltani Howyzeh et al., 2018), and *Echinophora tenuifolia* (Şanli et al., 2016 *)*, followed the same pattern and produced different essential oil content at various growth stages. This can be due to different physiological activities of these plants at various stages of development and its interaction with environment condition. These plants had low essential oil content in early growth stages, while the highest essential oil content was obtained at seed setting and seed phase. Less moisture content and lower activity of some essential enzymes which are necessary for biosynthesis of specific compounds at seeding stage lead to less essential oil production at early stages of growth (Hazrati et al., 2019; Şanli et al., 2016; Soltani Howyzeh et al., 2018). Furthermore, previous studies showed essential oil content was not the same in different plant organs. Results of the study on *H. persicum* showed 0.4 to 5.2% difference in essential oil content of various organs such as stem and seeds (Sefidkon et al., 2004). In our experiment, we observed a low content of the essential oil in vegetative and flowering stages and high essential oil content in seeds at setting stage. According to this, we can conclude that possibly, *H. persicum* plants spend most of their produced photosynthetic materials to create vegetative organs instead of synthesis of useful biological active compounds at the early stages of its growth in comparison to later stages.

**Figure 3:**
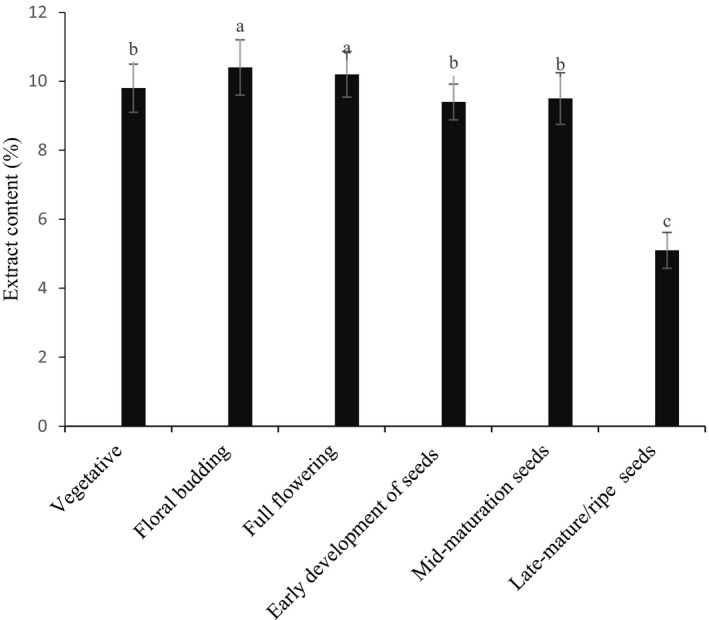
Changes in extract content (% w/w) of *H. persicum* at different phenological stages. Essential oil content with different letters was significantly different at *p* < .05 (*Tukey's* test)

Figure 3 shows *H. persicum* extracts content in different growing stages. According to our results, there was a significant difference between the extracts content in different phenological stages and the maximum extract content percentage was obtained in the floral budding stage (10.4%) followed by full flowering stage (10.2%). However, with the plant reaching the seed setting stage the extracts percentage decreased, as the lowest percentage of extracts were obtained at mature seeds stage at the rate of 5.1%. Based on the results, there were no significant differences between the flowering and floral budding stages. The situation was the same between vegetative and mid‐mature seeds stages.

### 3.2 Chemical compounds of essential oil

Recent reports suggest that biological features of the *H. persicum* such as anti‐inflammatory, anti‐pain, antioxidant, and anti‐seizure can be attributed to basic essential oil compounds (Majidi & Sadati Lamardi, 2018). The analysis of our data at various phenological stages of *H. persicum* indicated significant variations in the type and percentage of essential oil constituents (Table 1). In total, in the vegetative stages, floral budding stage, full flowering stage, early development of seeds stage, mid‐mature seeds stage, and final stage or late‐mature/ripe seeds stage, values of 96.8%, 97.4%, 96.8%, 98.1%, 97.1%, and 98.5% of the total essential oil constituent were, respectively, identified.

**Table 1:** Essential oil composition of *H. persicum* at different phenological stages and their mean comparisons

No.	Compounds	RI	Vegetative stage	Floral budding	Full flowering	Early development of seeds	Mid‐maturation of seeds	Late‐mature/ripe of seeds
1	Isopropyl isovalerate	894	0.4 ± 0.0 ^b^	tr	tr	tr	tr	1.6 ± 0.2^a^
2	α‐Pinene	930	1.9 ± 0.1 ^b^	4.5 ± 0.3 ^a^	4.9 ± 0.2 ^a^	2.3 ± 0.3 ^b^	tr	0.3 ± 0.0 ^c^
3	Isopropyl 3‐methyl−2‐butenoate	946	tr	tr	tr	tr	tr	1.2 ± 0.2
4	β‐Pinene	985	8.7 ± 0.2 ^a^	1.4 ± 0.1 ^b^	tr	tr	tr	0.3 ± 0.0 ^c^
5	Pseudolimonene	992	tr	2.2 ± 0.3 ^b^	3.0 ± 0.1 ^a^	0.5 ± 0.1 ^c^	tr	tr
6	Octanal	1,002	tr	0.3 ± 0.0 ^c^	0.3 ± 0.0 ^c^	tr	1.2 ± 0.1 ^a^	1.0 ± 0.1 ^b^
7	Butyl butyrate	1,005	tr	tr	tr	1.8 ± 0.1 ^b^	4.2 ± 0.7 ^a^	4.4 ± 0.4 ^a^
8	*p*‐Cymene	1,010	tr	1.4 ± 0.1 ^b^	4.2 ± 0.3 ^a^	tr	tr	tr
9	Limonene	1,018	18.1 ± 1.7 ^a^	0.5 ± 0.1 ^b^	tr	tr	tr	tr
10	Hexyl acetate	1,025	tr	tr	tr	0.4 ± 0.0 ^b^	0.9 ± 0.1 ^a^	1.0 ± 0.1 ^a^
11	Carene	1,027	4.2 ± 0.2 ^a^	1.2 ± 0.1 ^c^	2.7 ± 0.2 ^b^	0.6 ± 0.1 ^d^	tr	tr
12	γ‐Terpinene	1,036	1.4 ± 0.1 ^c^	4.4 ± 0.2 ^b^	6.3 ± 0.8 ^a^	1.0 ± 0.2 ^cd^	tr	0.7 ± 0.1 ^d^
13	Butyl 2‐methylbutanoate	1,044	tr	tr	tr	1.1 ± 0.1 ^a^	0.6 ± 0.1 ^b^	0.7 ± 0.1 ^b^
14	2‐Methylbutyl isobutyrate	1,048	tr	tr	tr	0.6 ± 0.1 ^a^	0.8 ± 0.1 ^a^	0.3 ± 0.0 ^b^
15	cis−5‐Octen−1‐ol	1,051	tr	tr	0.9 ± 0.0 ^c^	5.8 ± 0.2 ^a^	6.0 ± 0.2 ^a^	4.9 ± 0.2 ^b^
16	Linalool	1,061	0.5 ± 0.1 ^d^	0.6 ± 0.1 ^cd^	0.9 ± 0.2 ^c^	3.7 ± 0.2 ^a^	0.9 ± 0.1 ^c^	1.9 ± 0.1 ^b^
17	Thujone	1,098	tr	tr	tr	tr	tr	1.4 ± 0.1
18	4‐Methylpentyl isobutyrate	1,109	tr	tr	2.0 ± 0.2 ^c^	4.9 ± 0.2 ^b^	3.6 ± 0.2 ^b^	8.2 ± 0.8 ^a^
19	Camphor	1,136	tr	tr	tr	tr	tr	1.6 ± 0.1
20	Hexyl butyrate	1,183	0.9 ± 0.0 ^e^	8.2 ± 0.3 ^d^	9.1 ± 0.7 ^d^	32.1 ± 2.5 ^b^	38.8 ± 2.9 ^a^	23.6 ± 1.6 ^c^
21	Octyl acetate	1,203	tr	0.7 ± 0.1 ^d^	3.3 ± 0.4 ^c^	11.7 ± 0.9 ^ab^	14.5 ± 1.7 ^a^	10.5 ± 1.1 ^b^
22	Anethole	1,220	0.9 ± 0.1 ^c^	14.6 ± 1.0 ^a^	8.1 ± 1.1 ^b^	1.3 ± 0.1 ^c^	2.0 ± 0.2 ^c^	tr
23	Hexyl 2‐methylbutyrate	1,234	0.3 ± 0.1 ^d^	1.4 ± 0.1 ^d^	3.2 ± 0.4 ^c^	5.8 ± 0.6 ^b^	4.2 ± 0.2 ^c^	8.0 ± 0.8 ^a^
24	Hexyl isovalerate	1,240	tr	0.3 ± 0.1 ^b^	1.1 ± 0.1 ^ab^	2.0 ± 0.7 ^a^	2.1 ± 0.1 ^a^	2.5 ± 0.3 ^a^
25	Octyl Isobutyrate	1,329	tr	1.9 ± 0.1 ^b^	1.5 ± 0.2 ^b^	4.5 ± 0.5 ^a^	6.0 ± 0.7 ^a^	6.3 ± 0.9^a^
26	Hexyl hexanoate	1,369	tr	0.6 ± 0.1 ^d^	0.9 ± 0.0 ^c^	3.1 ± 0.4 ^ab^	3.7 ± 0.2 ^a^	2.6 ± 0.2 ^ab^
27	Octyl 2‐methylbutyrate	1,416	tr	0.7 ± 0.0 ^c^	1.1 ± 0.2 ^bc^	1.9 ± 0.1 ^b^	2.2 ± 0.2 ^b^	5.4 ± 0.7 ^a^
28	Caryophyllene	1,421	14.1 ± 1.5 ^a^	4.4 ± 0.2 ^b^	0.5 ± 0.1 ^c^	0.7 ± 0.1 ^c^	tr	0.9 ± 0.1 ^c^
29	Octyl isovalerate	1,442	tr	tr	0.3 ± 0.0 ^d^	0.4 ± 0.1 ^c^	0.7 ± 0.1 ^b^	1.1 ± 0.1 ^a^
30	α‐Curcumene	1,460	3.5 ± 0.4 ^b^	7.6 ± 0.5 ^a^	1.2 ± 0.1 ^c^	tr	tr	tr
31	Phenethyl 2‐methylbutyrate	1,481	1.4 ± 0.1 ^a^	0.9 ± 0.1 ^b^	1.3 ± 0.1 ^a^	tr	tr	tr
32	Myristicin	1,491	5.2 ± 0.7 ^b^	7.3 ± 0.6 ^b^	15.0 ± 1.3 ^a^	tr	tr	tr
33	(E)‐γ‐Bisabolene	1501	2.0 ± 0.7 ^a^	0.8 ± 0.1 ^b^	tr	tr	tr	tr
34	1,5,9,9‐Tetramethyl−1,4,7‐cycloundecatriene	1508	1.7 ± 0.1 ^a^	0.2 ± 0.1 ^c^	tr	tr	0.7 ± 0.1 ^b^	2.0 ± 0.2 ^a^
35	β‐Bisabolene	1516	8.6 ± 0.6 ^b^	12.6 ± 0.6 ^a^	8.3 ± 0.9^b^	1.7 ± 0.2 ^c^	tr	0.4 ± 0.1 ^c^
36	Spatulenol	1541	5.8 ± 0.3 ^a^	1.0 ± 0.3 ^b^	0.3 ± 0.0 ^c^	tr	tr	tr
37	1‐Allyl−2,3,4,5‐tetramethoxybenzene	1568	0.7 ± 0.1 ^b^	tr	3.0 ± 0.3 ^a^	tr	tr	tr
38	Caryophyllene oxide	1576	6.1 ± 0.3 ^a^	2.5 ± 0.3 ^b^	0.4 ± 0.1 ^d^	0.5 ± 0.1 ^d^	tr	0.8 ± 0.1 ^c^
39	d‐Viridiflorol	1591	tr	tr	tr	3.0 ± 0.4 ^b^	1.3 ± 0.1 ^c^	3.8 ± 0.2 ^a^
40	Butylphosphonic acid, hexyl 4‐methoxybenzyl ester	1597	0.3 ± 0.1 ^d^	1.9 ± 0.1 ^c^	2.8 ± 0.1 ^b^	3.6 ± 0.4 ^a^	1.4 ± 0.2 ^c^	tr
41	Apiol	1675	1.6 ± 0.1 ^c^	3.1 ± 0.3 ^b^	7.1 ± 0.9 ^a^	tr	tr	tr
42	1‐Tetradecanol	1681	7.1 ± 0.7 ^a^	5.4 ± 0.3 ^b^	2.4 ± 0.5 ^c^	0.7 ± 0.1 ^d^	tr	tr
43	Falcarinol	2005	0.4 ± 0.1 ^c^	2.6 ± 0.2 ^a^	0.7 ± 0.1 ^c^	1.4 ± 0.1 ^b^	tr	tr
44	Manool	2056	tr	0.4 ± 0.0 ^b^	tr	1.0 ± 0.1 ^a^	1.3 ± 0.1 ^a^	1.1 ± 0.1 ^a^
45	trans‐Geranylgeraniol	2,201	1.0 ± 0.5 ^ab^	1.8 ± 0.1 ^a^	0.4 ± 0.1 ^c^	tr	tr	tr
	Total		96.8	97.4	96.8	98.1	97.1	98.5

Note

Values are given as mean ± *SE* (*n* = 3). According to the Tukey's test application: means of the same column and main variable labeled with the same letters are not significantly different at *p* < .05.

The main constituents of the essential oil in various stages were limonene (18.1%), caryophyllene (14.1%), β‐bisabolene (8.9%), β‐pinene (8.7%), 1‐tetradecanol (7.1%), caryophyllene oxide (6.1%), spatulenol (5.8%), myristicin (5.2%), carene (4.2%), α‐curcumene (3.5%) at the vegetative stage. Anethole (14.6%), β‐bisabolene (12.6%), hexyl butyrate (8.2%), α‐curcumene (7.6%), myristicin (7.3%), 1‐tetradecanol (5.4%), α‐pinene (4.7%), γ‐terpinene (4.4%), caryophyllene (4.4%), and apiol (3.1%) were the main constituents of the essential oil in the early flowering stage. In the full flowering stage, values were as follows, myristicin (15.0%), hexyl butyrate (9.1%), β‐bisabolene (8.3%), anethole (8.1%), apiol (7.1%), γ‐terpinene (6.3%), α‐pinene (4.9%), p‐cymene (4.2%), octyl acetate (3.3%), and hexyl isovalerate (3.2%). During seed development, butyrate (32.1%), octyl acetate (11.7%), hexyl 2‐methyl butyrate (5.8%), cis‐5‐octen‐1‐ol (5.8%), 4‐methyl pentyl isobutyrate (4.9%), octyl isobutyrate (4.5%), and hexyl 4‐methoxybenzyl ester (3.6%) were dominant constituent of the essential oil. Lastly, in the phenological stage of immature seeds, hexyl butyrate (38.8%), octyl acetate (14.5%), cis‐5‐octen‐1‐ol (6.3%), hexyl 2‐methyl butyrate (4.2%), and 4‐methyl pentyl isobutyrate (3.6%) were measured as the predominant constituent of the essential oil; however, these percentages changed to 23.6, 10.5, 8.0, 4.8, and 8.2% at the seed final maturation stage, respectively (Table 1). Previous studies have shown almost the same compounds of the essential oil in *H*. *persicum's* plant seeds while the value of these compounds were negligible in the leaf and flower organs (Davari & Ezazi, 2017; Radjabian et al., 2014; Scheffer et al., 1984; Sefidkon et al., 2004). According to the mean comparisons’ analysis, there were significant differences between these compounds gathered in the various phenological stages (Table 1). The essential oil constituent was different in different phenological stages. In addition, predominant compounds of essential oil changed during different growth stages, which are also presented in the Table 1.

The study of essential oil constituents indicated that α‐pinene and β‐pinene were predominant in the early growth stages. According to the results (Table 1), the highest α‐pinene value was observed at the full flowering stage (4.9%), but we did not see the same results at the immature seed stage. Moreover, β‐pinene was detected in the vegetative stage and at the time of flower opening. With entering into the seed set stage, values of these two compounds decreased significantly.

Limonene was observed only in the vegetative and floral budding stages, and the highest value was 18.1% that obtained in the vegetative stage. In line with these results, other researchers reported the existence of this compound in the vegetative organs of *H. persicum* (Sefidkon et al., 2004), while it was rarely seen in the seeds. In the few cases that limonene was observed in seeds, the value was not significant.

P‐cymene was identified in the floral budding and full flowering stage, however its maximum value (4.2%) was observed in the full flowering stage. γ‐terpinene was detected in the full flowering stage and the floral budding stage (6.3 and 4.4%, respectively). In a study on the Ajowan plant, the amount of γ‐terpinene in the early stages of growth was low, and arrived to its maximum at flowering stage, then subsequently decreased, which could be due to the adsorption of pollinators (Soltani Howyzeh et al., 2018). α‐curcumene was observed only in the early growth stages of *H. persicum* and the maximum amount was related to the floral budding stage (7.6%). Caryophyllene and myristicin were the predominant compounds in the early growth period and full flowering which were quantified by the amount of 14.1 and 15.0%, respectively. β‐bisabolene was the predominant compound that detected in the floral budding stage and as the plant age increased, its value decreased.

Anethole was the predominant compounds in the floral budding stage and full flowering stage, and the highest value was obtained in the floral budding stage (14.6%); prior to flowering, its amount was not significant, and after that it greatly reduced. Apiol and 1‐tetradecanol were the other compounds that observed in the first three stages of plant vegetative stages. As shown in Table 1, the maximum values of apiol (7.1%) and 1‐tetradecanol (7.7%) belongs to the full flowering stage and the vegetative stages, respectively. Caryophyllene oxide was the compounds that declined with the increase in plant age, and the highest value was obtained in the vegetative stage at the rate of 6.1%.

The highest value of hexyl butyrate and octyl acetate, that are the most predominant and important compounds in *H. persicum*, was observed in the mid‐mature seeds stage, by 38.8% and 14.5%, respectively. These beneficial compounds significantly increased to the seedling stage. In agreement with our results, previous studies reported these two compounds as the predominant compound in the seeds of *H. persicum* (Majidi & Sadati Lamardi, 2018; Radjabian et al., 2014; Sefidkon et al., 2004).

Among all the compounds, hexyl 2‐methylbutyrate, octyl isobutyrate, and 4‐methylpentyl isobutyrate contents were increased with increase in plant age, so that at the end of the phonological stages, they were the dominant compounds.

**Figure 4:**
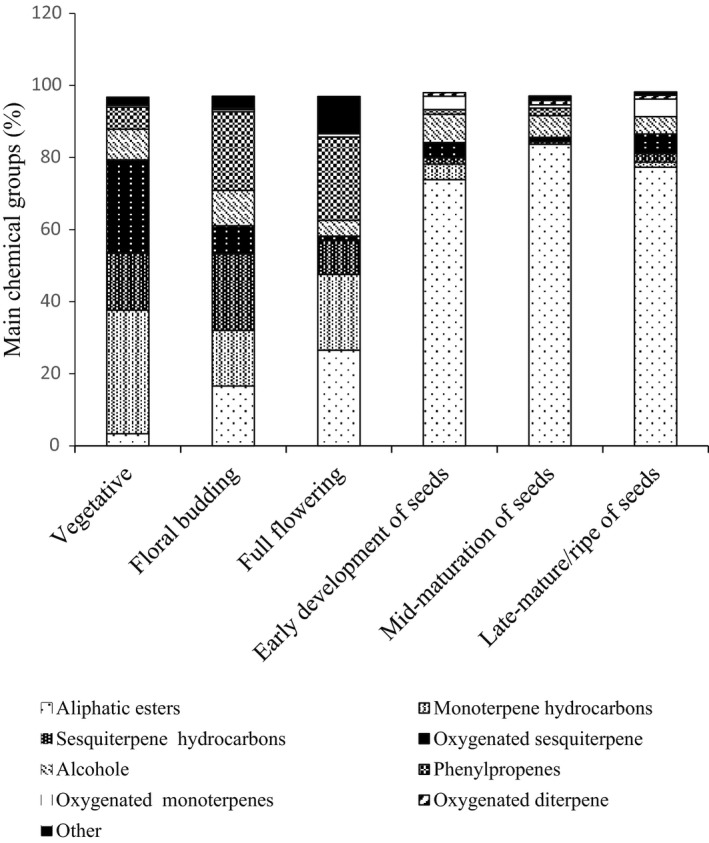
Comparison of main chemical groups (%) of *H. persicum* at different phenological stages

The essential oils were classified according to their chemical formula to eight groups (Figure 4). Based on the results, the main percentage of the essential oil was aliphatic esters, which value was 3.4% in the vegetative stage and finally hit 83.7% in the immature seeds stage or in the mid‐mature seeds stage. Therefore, by increasing the age of the plant and seeds formation, the value of this group of compounds was increased (Figure 4). The main components of aliphatic esters group were reported as hexyl butyrate, octyl acetate, octyl butyrate, and hexyl 2‐methylbutyrate, which their amounts were different in various phenological stages of growth. In the present study, these compounds were observed mostly in the last three phenological postflowering stages (Figure 4); other researchers also found more aliphatic compounds in the generative organs, such as in seeds of *H*. *persicum* (Boiss et al., 2014; Hajhashemi et al., 2009; Radjabian et al., 2013, 2014; Scheffer et al., 1984; Sefidkon et al., 2004).

The second and large group of the constituent compounds was the monoterpene hydrocarbons which their value in the early stage of the vegetative period was 34.2%, and with increasing the plant age and seed maturation, its value reduced, and finally this compound was not found in the mid‐mature seed stage (Figure 4). Several compounds of approximately 40 compounds belongs to monoterpene hydrocarbons group have been discovered by various researchers in the *H. persicum* (Amanpour et al., 2016; Boiss et al., 2014). In the present study, most of these compounds were observed in the flowering stage and prior to it, and when the plant entered into the seed set stage, the value of these compounds was significantly reduced (Figure 4). The most important compounds in this group were limonene, β‐pinene, α‐pinene, and γ‐terpinene. Other studies also confirmed these compounds as predominant compounds in the *H. persicum* (Mojab & Nickavar, 2003; Radjabian et al., 2014; Sefidkon et al., 2004). Similar to the present study, other researchers reported high amount of these compounds in flowers and leaves, in comparison to seeds (Mojab & Nickavar, 2003; Sefidkon et al., 2004;).

Sesquiterpene hydrocarbons were one of the other groups of the constituent compounds, and its amount was high at the beginning of the phenological growth time; the highest value was observed at the beginning stage of flowering (21.2%) while its value decreased with increasing plant age. Oxygenated sesquiterpene was another group of the constituent compounds. Analysis of our data showed 26.0% of sesquiterpene hydrocarbons at the vegetative stage; after this phenological stage, this amount significantly reduced (Figure 4). The most important compounds of this group were caryophyllene, spatulenol, and β‐bisabolene which also reported as predominant compounds in the *H. persicum* (Firuzi et al., 2010; Moshafi et al., 2009) in other studies.

The other compound evaluated in this experiment was phenylpropenes, which amount at the full flowering stage was 23.1%, and after passing this phenological stage it was significantly reduced (Figure 4). Anethole and myristicin were two important compounds related to phenylpropenes group. In a study on *H. persicum* plants, researchers found three phenylpropenes compounds (anethole, myristicin, and estragole) that were usually predominant in the vegetative and flower organs that match the results of the present study (Firuzi et al., 2010; Radjabian et al., 2013; Sefidkon et al., 2004).

Changes in essential oil compounds in different phenological stages of *H. persicum* growth are probably due to the fact that the production of essential oil and aromatic compounds are under the control of interactions between physiological, biochemical, and metabolic mechanisms which they depend on age and growth stages of plant. Besides, these changes related to terpene biosynthesis as well as its accumulation in the secretory organs (Németh, 2005). The change in the essential oil ingredients is also influenced by factors such as the age and the development stage of medicinal plants.

### 3.3 Phenolic acids in different phenological stages

Phenolic compounds such as phenolic acids are one of the most critical compounds in medicinal plants and they have paramount importance due to their high biological activity and function as antioxidants, anti‐inflammatory, anticancer, and anti‐Alzheimer (Goleniowski et al., 2013; Saibabu et al., 2015).

In this study, we investigated the phenolic acids content in different phenological stages of *H. persicum*. According to the obtained results, there were 8, 9, 9, 10, ,8 and 7 different phenolic compounds at the vegetative stages, floral budding stage, full flowering stage, early development of seeds stage, mid‐mature seeds stage and, final stage or late‐mature/ripe seeds stage, respectively (Table 2).

**Table 2:** Contents of phenolic acid compounds (mg/g dried extract) of *H. persicum* at different phenological stages

Phenological stage	GA	PHBA	VA	CaA	PCA	FA	MCA	CiA	RA	SA	Total
Vegetative	1.3 ± 0.5c	14.4 ± 0.2b	0.5 ± 0.0bc	0.0 ± 0.0c	13.4 ± 0.1c	9.9 ± 0.2c	0.0 ± 0.0c	16.1 ± 0.2c	13.3 ± 0.4a	3.6 ± 0.2a	72.5
Floral budding	1.9 ± 0.1c	8.6 ± 0.5c	0.6 ± 0.0b	0.0 ± 0.0c	24.1 ± 0.3b	12.0 ± 0.3b	4.9 ± 0.1b	225.3 ± 5.3a	9.2 ± 0.1b	1.0 ± 0.1b	287.6
Full flowering	4.0 ± 0.1b	16.8 ± 0.7a	8.3 ± 0.2a	0.0 ± 0.0c	39.2 ± 1.0a	15.8 ± 0.4a	6.4 ± 0.2a	56.4 ± 2.5b	6.3 ± 0.6c	0.7 ± 0.0b	153.9
Early development	1.3 ± 0.1c	3.6 ± 0.3d	0.6 ± 0.1b	5.6 ± 0.3a	11.9 ± 0.6cd	6.5 ± 0.3c	5.9 ± 0.4a	218.6 ± 5.7a	4.8 ± 0.5cd	0.9 ± 0.1b	259.7
Mid‐maturation	2.0 ± 0.1c	1.8 ± 0.1e	0.0 ± 0.0c	3.0 ± 0.3b	10.0 ± 0.9d	8.8 ± 0.5c	6.6 ± 0.2a	8.0 ± 0.3c	6.0 ± 0.5c	0.0 ± 0.0c	46.2
Late‐mature/ripe	6.5 ± 0.3a	0.8 ± 0.1e	0.0 ± 0.0c	0.0 ± 0.0c	1.7 ± 0.1e	2.4 ± 0.1d	0.0 ± 0.0c	8.7 ± 0.4c	3.6 ± 0.1d	1.1 ± 0.2b	24.8
Significance	**	**	**	**	**	**	**	**	**	**	

Note

Abbreviations: CaA, Caffeic acid; CiA, Cinnamic acid; FA, Ferulic acid; GA, Gallic acid; MCA, m‐Coumaric acid; PCA, p‐Coumaric acid; PHBA, p‐Hydroxybenzoic acid; RA, Rosmarinic acid; SA, Salicylic acid; VA, Vanillic acid.

Values are given as mean ± SE (*n* = 3). According to the *Tukey's* test application: means of the same column and main variable labeled with the same letters are not significantly different at *p* < .05.

The methanolic extract gathered from the floral budding stage had the highest amount of phenolic acids (287.6 mg/g dried extract) following by early seed development (259.7 mg/g dried extract), full flowering stage (153.9 mg/g dried extract), vegetative stage (72.5 dried mg/g extract), and mid‐mature seed stage (46.2 mg/g dried extract). On the other hand, the mature seed extract contained the minimum amount of phenolic acids (24.8 mg/g dried extract) (Table 2).

Cinnamic acid, p‐coumaric acid, p‐hydroxybenzoic acid, ferulic acid, and rosmarinic acid are predominant phenolic acids in *H. persicum*. Table 2 shows the different phenolic compounds at different morphological stages of *H. persicum* plants. The predominant phenolic acids were, cinnamic acid (16.1 mg/g extract), p‐hydroxybenzoic acid (14.4 mg/g extract), p‐coumaric acid (13.4 mg/g extract) in the vegetative stage, and rosmarinic acid (13.3 mg/g extract); in the floral budding stage, cinnamic acid (225.3 mg/g extract), p‐coumaric acid (24.1 mg/g extract), and ferulic acid (12.0 mg/g extract); in the full flowering stage, cinnamic acid (56.4 mg/g extract), p‐coumaric acid (39.2 mg/g extract), p‐hydroxybenzoic acid (16.8 mg/g extract); in the early stage of seed development, cinnamic acid (218.6 mg/g extract), p‐coumaric acid (11.9 mg/g extract), and ferulic acid (6.5 mg/g extract); in the mid‐mature seeds stage p‐coumaric acid (10.0 mg/g extract), ferulic acid (8.8 mg/g extract), and cinnamic acid (8.0 mg/g extract) and lastly in the final stage or late‐mature/ripe seeds stage, cinnamic acid (8.7 mg/g extract), Gallic acid (6.5 mg/g extract), and rosmarinic acid (3.6 mg/g extract).

Cinnamic acid was recorded as the predominant compounds of phenolic acid in all growth stages. However, its maximum value (225.3 mg/g extract) was in the early stages of flowering, and its lowest value (8.0 mg/g extract) was in the mid‐mature stage. Cinnamic acid is a natural aromatic phenolic acid whose long‐term consumption is associated with low toxicity to humans and is used in flavorings, artificial color, and some specific medications. The frequent use of cinnamic acid is as a precursor for the methyl cinnamate, ethyl cinnamate, and benzyl cinnamate production which have perfume industrial use. It is also a precursor for the artificial sweetener aspartame. Cinnamic acid is one of the phenolic acids with several biological and medicinal properties, as well as other economical and industrial values (Bradley et al., 2015; Goleniowski et al., 2013). According to our results, *H. persicum* is considered as one of the plants with a rich source of cinnamic acid, especially in the floral budding stage and early development of seeds stage; however, as the plant entered the mature seed stage, we observed the significant reduction in its value.

P‐coumaric acid was another beneficial predominant phenolic acid we explored in this experiment. P‐coumaric acid amount was increased during the plant growth to 39.2 mg/g at the full flowering stage and then decreased to the lowest amount in the seed maturing stage (1. 7 mg/g extract). Studies showed p‐coumaric acid has antioxidant properties, which can reduce the stomach cancer by suppressing nitrous amines, and has anti‐tumor and anti‐mutagenesis activities (Goleniowski et al., 2013; Taofiq et al., 2017).

Ferulic acid is one of the other critical phenolic compounds that researchers have been proven its antioxidant properties (Goleniowski et al., 2013). This compound was found at all growth stages of the *H. persicum* plant, and the highest and lowest amount were recorded for the full flowering stage and the seed mature stage with values 15.8 and 2.4 mg/g, respectively.

P‐hydroxybenzoic acid is noted as the basis for the preparation of its esters, named parabens, and they are employed as preservatives in cosmetics and some ophthalmic solutions. This material was one of the other predominant phenolic acids in the *H. persicum* observed in all phenological stages in this experiment. According to the obtained results, the maximum value of 16.8 mg/g extract was obtained at the flowering stage, while the seed maturing stage had the lowest amount (0.8 mg/g extract). The amount of this phenolic acid decreased significantly by entering the seed into the sowing stage. Rosmarinic acid is an ester of caffeic acid called 3,4‐dihydroxy phenyl lactic acid (Goleniowski et al., 2013; Lamaison et al., 1991). This compound has many pharmaceutical properties such as antimicrobial, anti‐rheumatism, and anticancer and was present in all phenological stages of the *H. persicum*. The highest (13.3 mg/g extract) and the lowest amounts (3.6 mg/g extract) of this compound were obtained in the vegetative stage and the immature seed stage, respectively. Therefore, it can be concluded that *H. persicum* can be considered as a plant rich in phenolic acids, which content was different in different phenological stages. Phenolic compounds were at the lowest rate in the floral budding stage while the maximum content was recorded at the seed mature stage. An increase in phenolic acid levels during flowering stage recommended a higher expression level of the phenylalanine lyase enzyme (Andreotti et al., 2006; Feduraev et al., 2019), and it is also an indication of enzyme activity reduction with plant maturation. These changes that occur in the process of primary metabolites adsorption are a result of starch synthesis in the middle stages of seed maturation, and this phenomenon can affect the biosynthesis of phenolic acids (Ma et al., 2016).

### 3.4 Cluster analysis and principal component analysis (PCA)

Another goal of this study was to monitor the differences and similarities between different phenological stages in order to find the consequences of different harvesting time on identified phytochemical compounds in *H. persicum*. The results of the main components analyses are shown in Table 3. Based on these results, three components had highest eigenvalue, reporting 89.45% of the total variance. The relative variance for the first, second, and third components was 55.52%, 23.28%, and 10.65%, respectively. In the first component, the compounds of butyl butyrate, carene, 4‐methylpentyl isobutyrate, hexyl butyrate, cis‐5‐octen‐1‐ol, octyl acetate, hexyl 2‐methylbutyrate, octyl isobutyrate, β‐bisabolene, 1‐tetradecicanol, p‐hydroxybenzoic acid, rosmarinic acid, and essential oil content had the highest loading factor. Differently, in the second and third components, m‐coumaric acid and gallic acid compounds had the most top loading factor, respectively (Table 3).

**Table 3:** Principal component analysis of main phytochemical compounds for different phenological stages of *H. persicum* medicinal plants

Number of compounds	Phytochemical compounds	Principal component (PC)		
		PC1	PC2	PC3
x_1_	a‐Pinene	−0.76	0.59	−0.02
x_2_	β‐Pinene	−0.61	−0.78	0.00
x_3_	Butyl butyrate	0.95	−0.15	0.13
x_4_	Limonene	−0.56	−0.79	0.02
x_5_	Carene	−0.87	−0.29	0.17
x_6_	γ‐Terpinene	−0.68	0.68	0.25
x_7_	4‐Methylpentyl isobutyrate	0.89	−0.01	0.37
x_8_	Hexyl butyrate	0.92	0.15	−0.32
x_9_	cis−5‐Octen−1‐ol	0.97	0.02	−0.16
x_10_	Anethole	−0.57	0.60	−0.19
x_11_	Octyl acetate	0.96	0.09	−0.15
x_12_	Hexyl 2‐methylbutyrate	0.89	0.14	0.32
x_13_	Octyl Isobutyrate	0.98	0.08	0.01
x_14_	a‐Curcumene	−0.72	−0.02	−0.27
x_15_	Caryophyllene	−0.67	−0.74	−0.05
x_16_	Myristicin	−0.77	0.53	0.32
x_17_	β‐Bisabolene	−0.94	0.14	−0.06
x_18_	Apiol	−0.69	0.62	0.34
x_19_	Caryophyllene oxide	−0.69	−0.72	−0.04
x_20_	1‐Tetradecanol	−0.91	−0.36	−0.12
x_21_	Gallic acid	0.36	0.14	0.89
x_22_	p‐Hydroxybenzoic acid	−0.91	0.13	0.21
x_23_	Vanillic acid	−0.42	0.68	0.43
x_24_	Caffeic acid	0.55	0.10	−0.66
x_25_	p‐Coumaric acid	−0.69	0.70	0.07
x_26_	Ferulic acid	−0.77	0.53	−0.09
x_27_	m‐Coumaric acid	0.08	0.80	−0.52
x_28_	Cinnamic acid	−0.17	0.37	−0.62
x_29_	Rosmarinic acid	−0.81	−0.51	−0.21
x_30_	Salicylic acid	−0.57	−0.77	0.11
x_31_	Essential oil content	0.81	−0.06	−0.06
x_32_	Extraction content	−0.65	0.30	−0.61
Eigenvalue		17.77	7.45	3.41
Relative variance (%)		55.52	23.28	10.65
Cumulative variance (%)		55.52	78.80	89.45

**Figure 5:**
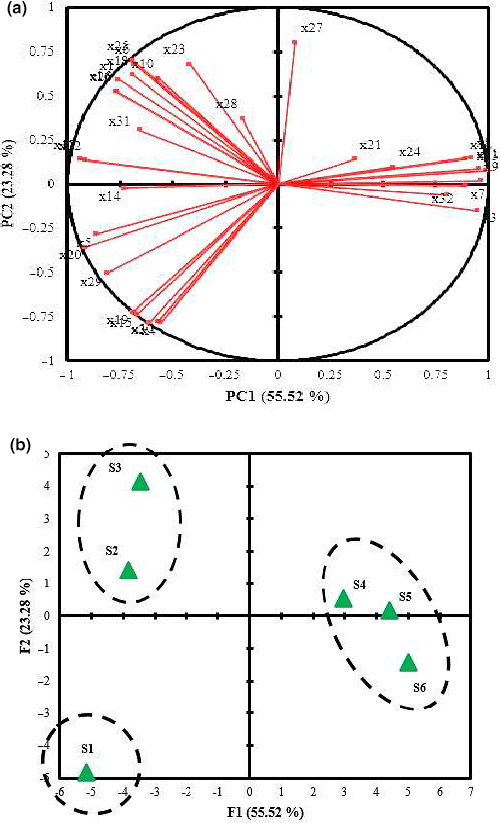
Biplot derived based on first and second principle components (PC) for different phenological stages of *H. persicum* medicinal plants

Since 78.80% of the total variance was allocated to the first and second components, they were used for the biplot diagram obtained from the PCA. Different phenological stages were divided into three distinct groups which shown in the Figure 5. The biplot analysis showed the vegetative stage (S1), based on the first and second components alone and with high distance with other phenological stages. It was strongly correlated with the compounds of carene, rosmarinic acid, anethole, caryophyllene, β‐pinene, limonene, and salicylic acid. The phenological stages of floral budding (S2) and full flowering (S3) were segregated in one group according to the first and second components which have a high correlation with β‐bisabolene, p‐hydroxybenzoic acid, anethole, p‐coumaric acid, γ‐terpinene, apiol, a‐pinene, myristicin, ferulic acid, extraction content, cinnamic acid and, vanillic acid compounds. On the other hand, other phenological stages, including early seed development, mid‐mature seed, and mature seed, were grouped together and have a strong correlation with butyl butyrate, 4‐methyl pentyl isobutyrate, hexyl 2‐methyl butyrate, hexyl butyrate, octyl acetate, cis‐5‐octen‐1‐ol, octyl isobutyrate, caffeic acid, gallic acid, and essential oil content. In research on the *Ferulago angulata*, using principal component analysis, the first two components reported as 76% of the total variance (Hazrati et al., 2019). Results of this study also showed that the measured phytochemical compounds were affected by different treatments. Moreover, they showed that for the first component, eight compounds had the highest loading factor. Esmaeili et al. (2018) showed the first two components accounted for 95% of the total variance using the principal component analysis based on phytochemical compounds of *Oliveria decumbens* (Esmaeili et al., 2018). They showed that 12 and 7 compounds accounted for the highest factor loading in the first and second components, respectively. Other researchers also used principal component analysis to identify the dominant compounds in the useful components in other medicinal plants like *Satureja pilosa* (Dardioti et al., 2012).

Considering the higher extract content quantity or quality in the general floral budding and full flowering stage, it is possible to point the suitability of harvest in these stages for producing a higher extract content. The final growth stage also had higher essential oil percentage, indicating the effect of different phenological stages on the essential oil percentage quality. So we can pinpoint the phytochemical compounds extracted from the plant strongly depend on the plant organ and phenological stage. (Kaškonienė et al., 2011; Özgüven & Tansi, 1998; Şanli et al., 2016).

In a study to investigate the phenological effects on phytochemical compounds at different populations of the *Tithonia diversifolia*, the cluster and principal component analyses were used and the results showed that phytochemical compounds were differed in various phenological stages (Pretti et al., 2018).

**Figure 6:**
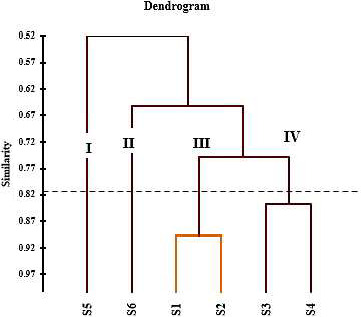
Hierarchical cluster analysis based on all studied traits in different phenological stages of *H. persicum* medicinal plants

Classified phenological stages based on all the studied characteristics in this experiment using cluster analysis method are shown in Figure 6. Based on the dendrogram obtained by cluster analysis, different phenological stages were segregated into four separate groups. Primary growth stages consisted of the vegetative stage (S1) and floral budding stage (S2) and they were more similar to each other in terms of characteristics. The middle phenological stages, including full flowering (S3) and early seed development (S4), were placed in a separate group, and the mid‐mature stage of seed (S5) and matured seed (S6) were individually grouped. Hazrati et al. (2019) used the cluster analysis method to classify the target treatments based on identified phytochemical compounds (Hazrati et al., 2019). The results of the cluster analysis based on different phenological stages showed that two primary and intermediate stages of physiological processes were highly correlated with each other, but were strongly influenced by the plant physiological stage while were separately placed in a separate group. Based on the dendrogram obtained from the cluster analysis, it was observed that the *H. persicum* was affected by the genetic and the environment in the final stages of phenology more than the primary and middle stages. Pretti et al. (2018) stated that principal component analysis and cluster analyses can be used to determine the optimum phenological stage and to achieve the best yield of phenolic acids and essential oil compounds from plants (Pretti et al., 2018). This research illustrated in the vegetative phase and in the beginning of the generative phase; the rate of phenolic acids was high. The current research confirmed that the different phenological stages had affected the phenolic acids contents and essential oil compounds of *H. persicum*. However, the mechanisms in which these changes have been made will require further studies.

## 4 CONCLUSION

*H. persicum* is one of the most important aromatic medicinal plants in Iran that has valuable phytochemicals compounds, which make it a precious medicinal plant to grow in other parts of the world. The results of this study clearly indicated that the yield and quality of the phytochemical composition of the *H. persicum* were significantly changed during the growth period and strongly depends on various phenological stages. The highest yield of essential oil was obtained in the intermediate stage of seed mature, and the highest content of extract was captured in the floral budding stage. Limonene, myristicin, and anethole were predominant compounds in vegetative and flowering stages, and the best stage for achieving the maximum value of hexyl butyrate and octyl acetate content was the seed formation stage. The results of the present study introduced *H. persicum* as a rich source of phenolic acids, of which cinnamic acid was one of the predominant compounds in all growing stages, and its highest value was obtained in the floral budding stage. Finally, it can be concluded that to reach the maximum essential oil content and composition of aliphatic esters compounds, harvest at the seed set stage, especially in the mid‐mature seeds stage, is desirable. In addition, harvesting the *H. persicum* at the flowering stage, especially at the floral budding stage, leads to maximum content of extract yield and phenolic acid compounds.

## 5 ETHICAL REVIEW

This study does not involve any human or animal testing.

## 6 INFORMED CONSENT

Written informed consent was obtained from all study participants.

## CONFLICT OF INTEREST

The authors declare that they do not have any conflict of interest.

## AUTHOR CONTRIBUTIONS

Saeid Hazrati, Seyyed Jaber Hosseini, and Silvana Nicola: were involved in designed and conducted the research, data collection, analysis of results, and writing‐original draft manuscript. Saeed Mollaei, Hossein Rabbi Angourani, Seyyed Jaber Hosseini, and Mojde Sedaghat: were involved in development of the study design and data curation. Saeid Hazrati, Seyyed Jaber Hosseini, and Saeed Mollaei: were involved in development of the analysis of results and contributed to submitting the manuscript. Saeid Hazrati, Mojde Sedaghat: and Silvana Nicola reviewed and revised the manuscript.

## Data Availability

Research data are not shared.
